# Genomic Analysis of a Strain Collection Containing Multidrug-, Extensively Drug-, Pandrug-, and Carbapenem-Resistant Modern Clinical Isolates of Acinetobacter baumannii

**DOI:** 10.1128/aac.00892-22

**Published:** 2022-08-15

**Authors:** Adam Valcek, Kristina Nesporova, Clémence Whiteway, Tim De Pooter, Wouter De Coster, Mojca Strazisar, Charles Van der Henst

**Affiliations:** a Microbial Resistance and Drug Discovery, VIB-VUB Center for Structural Biology, VIB, Flanders Institute for Biotechnology, Brussels, Belgium; b Structural Biology Brussels, Vrije Universiteit Brussel (VUB), Brussels, Belgium; c Neuromics Support Facility, VIB Center for Molecular Neurology, VIB, Antwerp, Belgium; d Department of Biomedical Sciences, University of Antwerp, Antwerp, Belgium; e Applied and Translational Neurogenomics Group, VIB Center for Molecular Neurology, VIB, Antwerp, Belgium; f Applied and Translational Neurogenomics Group, Department of Biomedical Sciences, University of Antwerp, Antwerp, Belgium

**Keywords:** *Acinetobacter baumannii*, bacteriology, microbiology, plasmids, whole-genome sequencing, antibiotic resistances

## Abstract

In this study, we characterize a new collection that comprises multidrug-resistant (MDR), extensively drug-resistant (XDR), pandrug-resistant (PDR), and carbapenem-resistant modern clinical isolates of Acinetobacter baumannii collected from hospitals through national microbiological surveillance in Belgium. Bacterial isolates (*n* = 43) were subjected to whole-genome sequencing (WGS), combining Illumina (MiSeq) and Nanopore (MinION) technologies, from which high-quality genomes (chromosome and plasmids) were *de novo* assembled. Antimicrobial susceptibility testing was performed along with genome analyses, which identified intrinsic and acquired resistance determinants along with their genetic environments and vehicles. Furthermore, the bacterial isolates were compared to the most prevalent A. baumannii sequence type 2 (ST2) (Pasteur scheme) genomes available from the BIGSdb database. Of the 43 strains, 40 carried determinants of resistance to carbapenems; *bla*_OXA-23_ (*n* = 29) was the most abundant acquired antimicrobial resistance gene, with 39 isolates encoding at least two different types of OXA enzymes. According to the Pasteur scheme, the majority of the isolates were globally disseminated clones of ST2 (*n* = 25), while less frequent sequence types included ST636 (*n* = 6), ST1 (*n* = 4), ST85 and ST78 (*n* = 2 each), and ST604, ST215, ST158, and ST10 (*n* = 1 each). Using the Oxford typing scheme, we identified 22 STs, including two novel types (ST2454 and ST2455). While the majority (26/29) of *bla*_OXA-23_ genes were chromosomally carried, all *bla*_OXA-72_ genes were plasmid borne. Our results show the presence of high-risk clones of A. baumannii within Belgian health care facilities with frequent occurrences of genes encoding carbapenemases, highlighting the crucial need for constant surveillance.

## INTRODUCTION

Acinetobacter baumannii (1) is a Gram-negative opportunistic pathogen, recognized as a problematic hospital pathogen often resistant to multiple antimicrobials, prolonged desiccation periods, disinfectants, and the immune system (2). Isolation of carbapenem-resistant A. baumannii (CRAb) is ranked as a top priority and urgent threat by the WHO and CDC, respectively. Beside its intrinsic resistances, A. baumannii is also capable of acquiring resistance to different clinically relevant antibiotics, limiting the therapeutic options and therefore leading to treatment failure (3). CRAb isolates have also been shown to increase globally, with rates reaching or exceeding 90% in some clinical settings in southern and eastern European countries and elsewhere (4). Mortality rates for the most common CRAb infections, such as bloodstream infections and hospital-acquired pneumonia, approach 60% (5). Most CRAb isolates harbor genes encoding acquired carbapenem-hydrolyzing class D β-lactamases and/or class B metallo-β-lactamases. Some CRAb isolates produce an intrinsic OXA-51-like carbapenemase, whereas others produce class B metallo-β-lactamases, including IMP and NDM types (6). The OXA-type carbapenemases represent the most prevalent mechanism of carbapenem resistance in this species, with OXA-23, -24, and -58 being the most prevalent (7). Between 2009 and 2011, OXA-23 producers emerged and replaced the previously predominant OXA-58-producing A. baumannii strains (8).

In this study, we sequenced and generated whole-genome *de novo* assemblies of a nonredundant collection of 43 new clinical isolates of A. baumannii that contain multidrug-resistant (MDR), extensively drug-resistant (XDR), pandrug-resistant (PDR), and carbapenem-resistant strains, along with performing genetic and phenotypic characterizations and comparisons.

## RESULTS AND DISCUSSION

**FIG 1:**
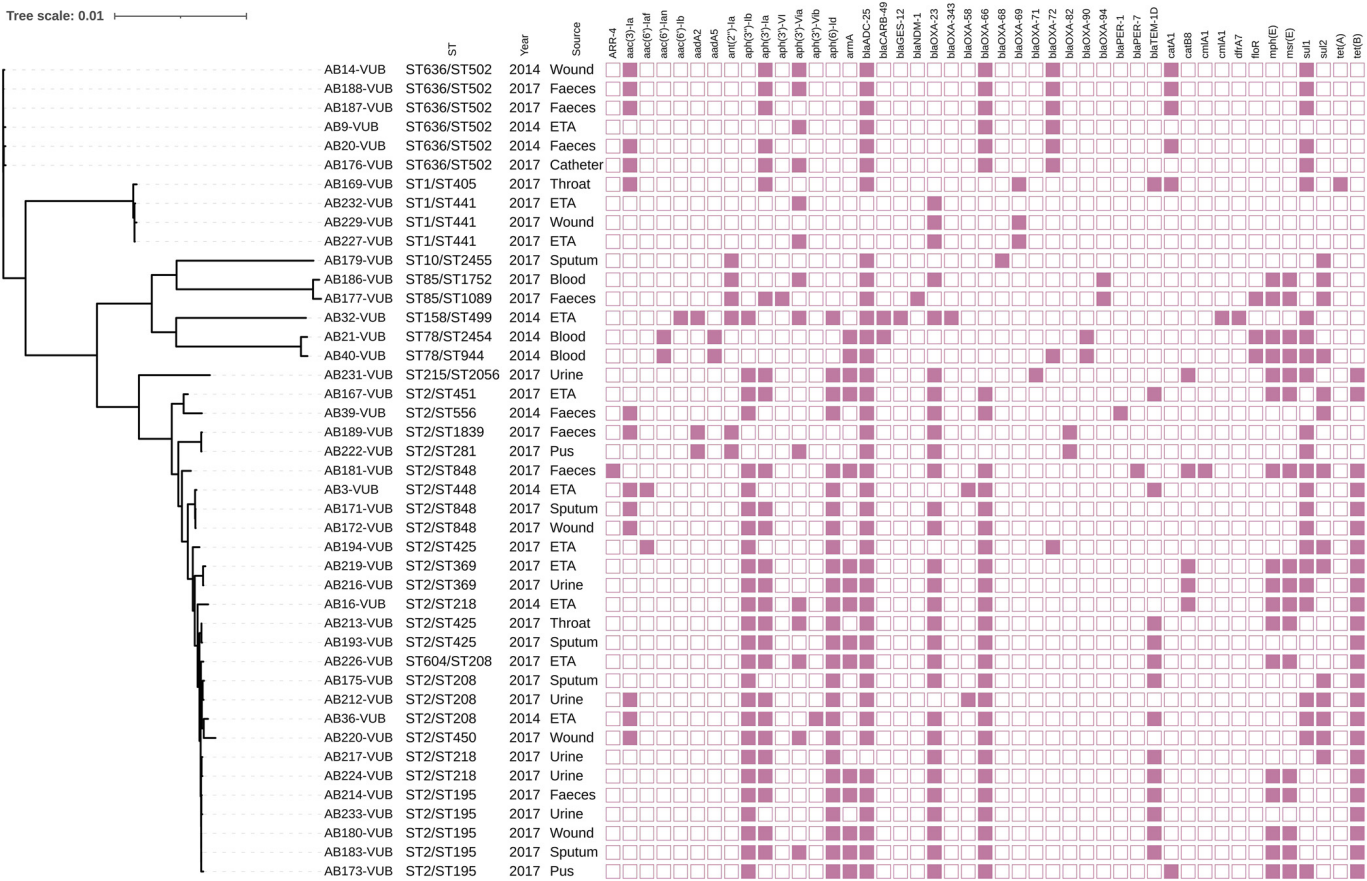
Phylogenetic tree of 43 clinical isolates of A. baumannii with depiction of the name, ST^Pasteur^/ST^Oxford^, year of isolation, source and resistance genes, respectively. ETA, endotracheal aspirate.

**FIG 2:**
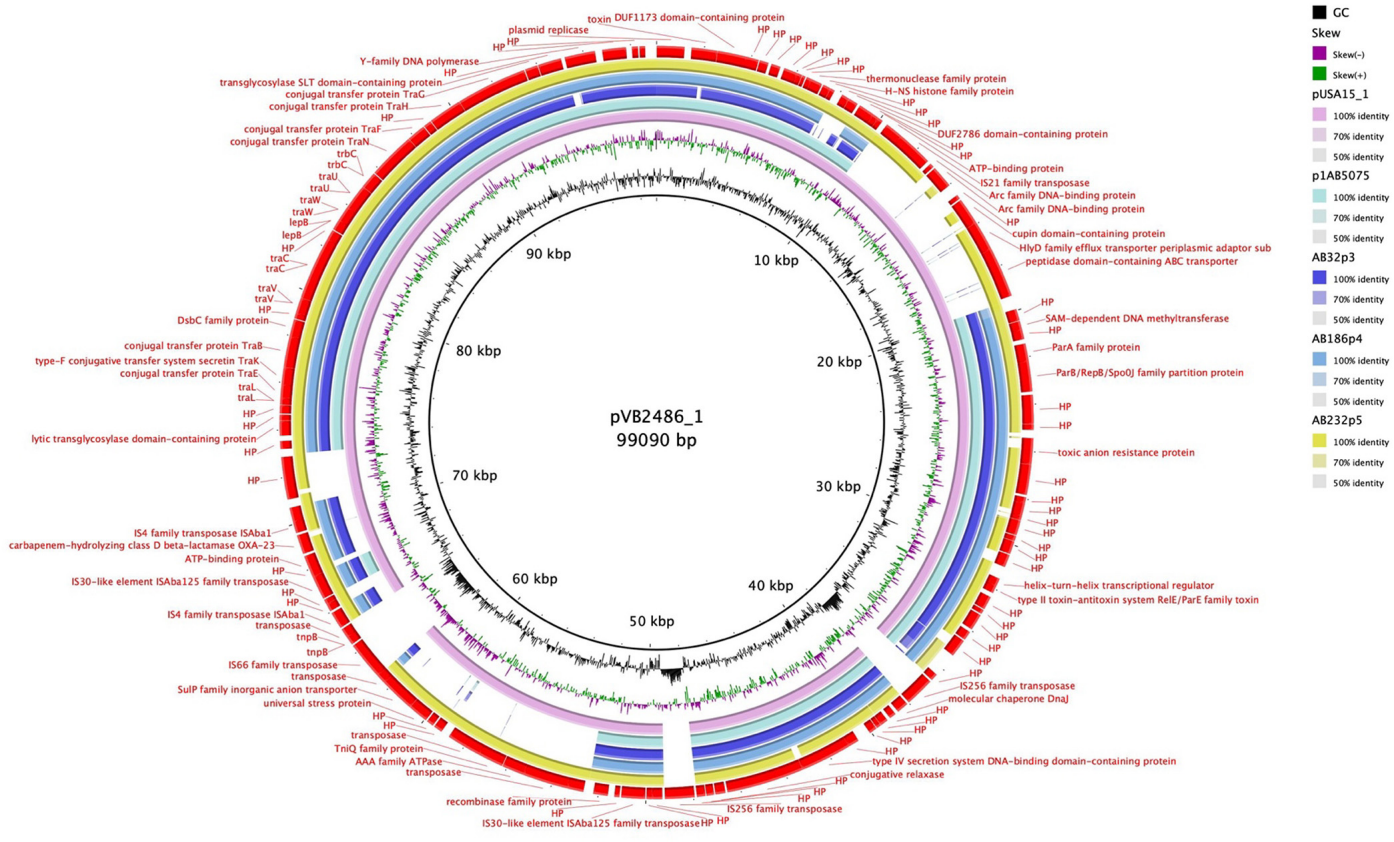
BLAST Ring Image Generator (BRIG) comparison of p3AB32-VUB, p4AB186-VUB, and p5AB232-VUB with pUSA15_1 and pVB2486_1 as a reference, showing a high nucleotide similarity of plasmids originating from Belgian isolates to pUSA15_1, pVB2486_1, and p5AB232-VUB of clinical origin.

**FIG 3:**
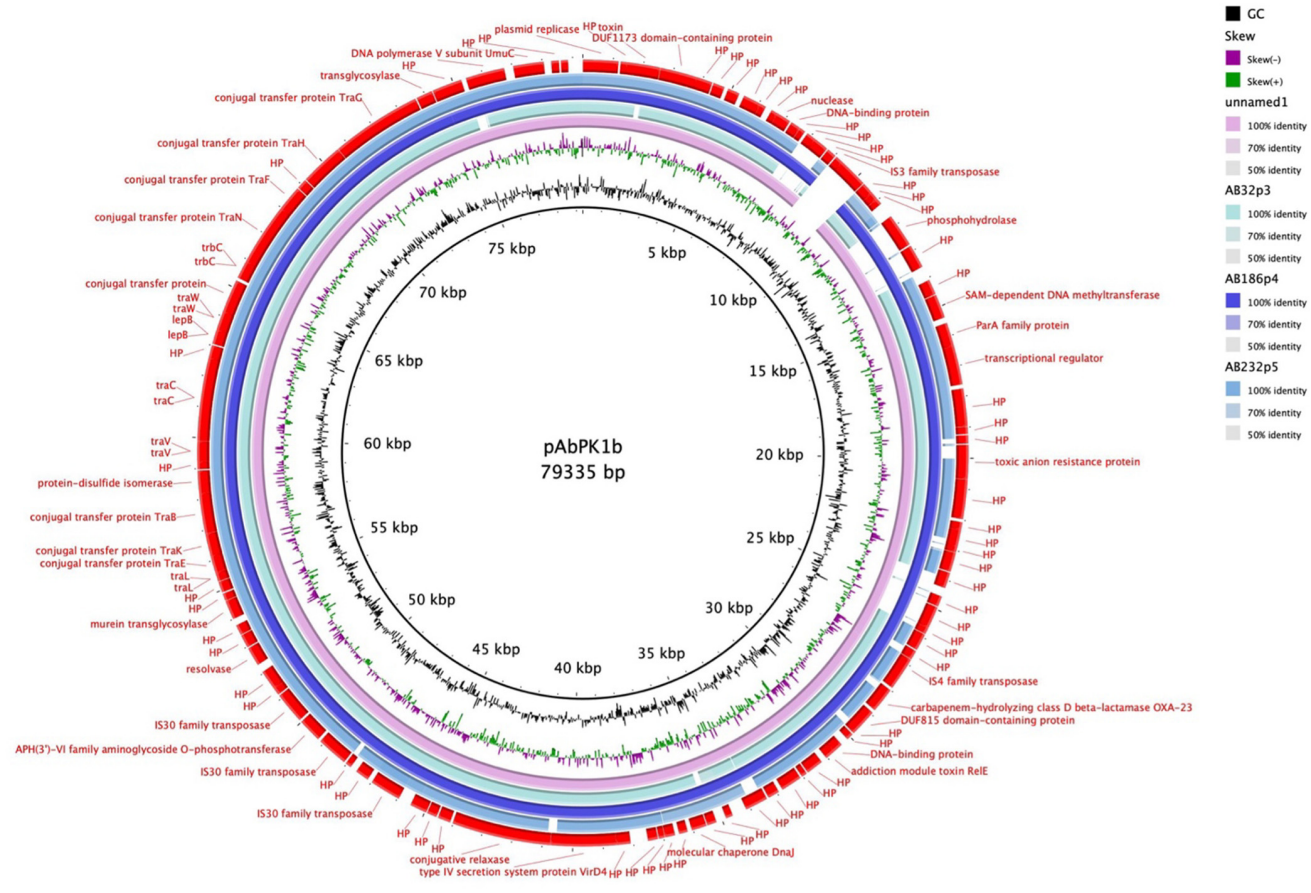
BRIG comparison of p3AB32-VUB, p4AB186-VUB, and p5AB232-VUB with unnamed1 and pAbPK1b as a reference, showing a high nucleotide similarity of plasmids originating from Belgian isolates to pAbPK1b of animal origin.

The clinical strain collection of this study contains 9 MDR, 24 XDR, and 10 PDR genotypes, all phenotypically confirmed (Fig. 1 and Table 1). Genotyping of the isolates showed *bla*_OXA-23_ (*n* = 29) as the most frequent acquired beta-lactamase gene, while the other acquired *bla* carbapenemase-encoding genes were *bla*_OXA-58_ (*n* = 2) *bla*_OXA-72_ (*n* = 8), and *bla*_NDM-1_ (*n* = 1). Intrinsic oxacillinase genes *bla*_OXA-66_, *bla*_OXA-68_, *bla*_OXA-69_, *bla*_OXA-71_, *bla*_OXA-72_, *bla*_OXA-82_, *bla*_OXA-90_, *bla*_OXA-94_, and *bla*_OXA-343_ were present, showing the significant variability among the clinical isolates in our collection. The majority (26/29) of *bla*_OXA-23_ genes lay within Tn*2006*, which is a mobile genetic element consisting of *bla*_OXA-23_ and two copies of IS*Aba1*, commonly associated with A. baumannii encoding OXA-23 (9, 10). Isolates AB32-VUB and AB186-VUB carried *bla*_OXA-23_ within Tn*2008* and harbored one copy of IS*Aba1* (Table 2). Isolates AB32-VUB, AB186-VUB, and AB232-VUB carried gene *bla*_OXA-23_ on large GR6 plasmids (Fig. 2 and 3). One of the plasmids from isolate AB232-VUB, designated p5AB232, shows high sequence similarity to pVB2486 (GenBank accession no. NZ_CP050404.1) and pUSA15_1 (NZ_CP020594) from clinical isolates from South Korea (isolated in 2013) and India (isolated in 2019), respectively (Fig. 2). However, the sequences of plasmids p3AB32-VUB and p4AB186-VUB resemble that of pAbPK1b (NZ_CP024578) from an isolate of a sheep origin from Pakistan, detected in 2012, and to “unnamed1” (NZ_CP069841.1) from clinical isolate FDAARGOS_1360 from the United States, detected in 2021.

**TABLE 1:** Isolates harboring acquired carbapenem resistance genes, their localization, and their genetic environment

Carbapenemase	Isolate	Gene localization	Associated mobile element
OXA-23	AB16-VUB	Chromosome	Tn*2006*
	AB32-VUB	Plasmid	Tn*2008*
	AB36-VUB	Chromosome	Tn*2006*
	AB39-VUB	Chromosome	Tn*2006*
	AB167-VUB	Chromosome	Tn*2006*
	AB171-VUB	Chromosome	Tn*2006*
	AB172-VUB	Chromosome	Tn*2006*
	AB173-VUB	Chromosome	Tn*2006*
	AB175-VUB	Chromosome	Tn*2006*
	AB180-VUB	Chromosome	Tn*2006*
	AB181-VUB	Chromosome	Tn*2006*
	AB183-VUB	Chromosome	Tn*2006*
	AB186-VUB	Plasmid	Tn*2008*
	AB189-VUB	Chromosome	Tn*2006*
	AB193-VUB	Chromosome	Tn*2006*
	AB213-VUB	Chromosome	Tn*2006*
	AB214-VUB	Chromosome	Tn*2006*
	AB216-VUB	Chromosome	Tn*2006*
	AB217-VUB	Chromosome	Tn*2006*
	AB219-VUB	Chromosome	Tn*2006*
	AB220-VUB	Chromosome	Tn*2006*
	AB222-VUB	Chromosome	Tn*2006*
	AB224-VUB	Chromosome	Tn*2006*
	AB226-VUB	Chromosome	Tn*2006*
	AB227-VUB	Chromosome	Tn*2006*
	AB229-VUB	Chromosome	Tn*2006*
	AB231-VUB	Chromosome	Tn*2006*
	AB232-VUB	Plasmid	Tn*2008*
	AB233-VUB	Chromosome	Tn*2006*
OXA-58	AB3-VUB	Plasmid	IS*Aba3* composite transposon
	AB212-VUB	Chromosome	IS*18*
OXA-72	AB9-VUB	Plasmid	IS*Aba31*
	AB14-VUB	Plasmid	IS*Aba31*
	AB20-VUB	Plasmid	IS*Aba31*
	AB40-VUB	Plasmid	ND^*a*^
	AB176-VUB	Plasmid	IS*Aba31*
	AB187-VUB	Plasmid	IS*Aba31*
	AB188-VUB	Plasmid	IS*Aba31*
	AB194-VUB	Plasmid	IS*Aba31*
NDM-1	AB177-VUB	Chromosome	IS*Aba125* composite transposon

a ND, not detected.

**TABLE 2:** Antibiotic resistance profiles of the Belgian clinical isolates^*a*^

	Resistance to:													
Strain	AMS	PIP	PIT	CAZ	ATM^*b*^	MEM	GEN	AMK	COL	CIP	TGC^*b*^	SXT	TET (Etest)	Phenotype
AB3-VUB	R	R	R	I+	S	R	R	S	I	R	S	R	R	PDR
AB9-VUB	NR	R	R	I+	I	R	R	R	R	R	S	S	NT	MDR
AB14-VUB	I	R	R	I+	I	R	R	R	R	R	S	R	S	XDR
AB16-VUB	R	R	R	I+	I+	R	R	R	R	R	S	R	R	PDR
AB20-VUB	NS	R	R	I+	I	R	R	R	NS	R	S	R	NT	XDR
AB21-VUB	NR	R	R	I+	I	S	R	R	NR	R	S	R	NT	MDR
AB32-VUB	R	R	R	I+	I+	R	R	R	S	R	S	R	NT	XDR
AB36-VUB	R	R	R	I+	I+	R	R	R	I	R	S	R	R	PDR
AB39-VUB	R	R	R	I+	I+	R	R	NR	NS	R	S	R	NT	XDR
AB40-VUB	NS	R	R	I+	I+	R	R	R	NR	R	S	R	NT	XDR
AB167-VUB	R	R	R	I+	I+	R	R	R	R	R	S	R	R	PDR
AB169-VUB	R	R	R	I+	I	S	R	S	I	R	S	R	NT	XDR
AB171-VUB	NS	R	R	I+	I	R	R	S	S	R	S	R	NT	XDR
AB172-VUB	NS	R	R	I+	I	R	R	S	S	R	S	R	NT	XDR
AB173-VUB	NR	R	R	I+	I	R	R	R	R	R	S	R	NT	XDR
AB175-VUB	R	R	R	I+	I	R	NR	S	NS	R	S	R	NT	XDR
AB176-VUB	R	R	R	I+	I+	R	R	NQ	I	R	S	R	NT	XDR
AB177-VUB	R	R	R	I+	I+	R	R	S	S	R	S	R	NT	XDR
AB179-VUB	S	R	R	I	I	S	S	S	I	S	S	S	NT	MDR
AB180-VUB	R	R	R	I+	I+	R	R	R	NR	R	S	S	NT	MDR
AB181-VUB	R	R	R	I+	I+	R	R	R	NS	R	S	R	R	PDR
AB183-VUB	R	R	R	I+	I+	R	R	R	I	R	S	S	NT	XDR
AB186-VUB	R	R	R	I+	I+	R	R	R	S	R	S	R	NT	XDR
AB187-VUB	NS	R	R	I+	I	R	R	R	R	R	S	R	NT	XDR
AB188-VUB	NQ	R	R	I+	I	R	R	NS	R	R	S	R	NT	XDR
AB189-VUB	R	R	R	I+	I+	R	R	S	NS	R	S	R	S	XDR
AB193-VUB	R	R	R	I+	I+	R	R	R	R	R	S	R	R	PDR
AB194-VUB	R	R	R	I+	NR	R	I	R	I	R	S	R	R	PDR
AB212-VUB	NR	R	R	I+	S	R	R	S	NR	R	S	R	NT	MDR
AB213-VUB	R	R	R	I+	I	R	R	R	R	R	S	R	R	PDR
AB214-VUB	R	R	R	I+	I+	R	R	R	NR	R	S	S	NT	MDR
AB216-VUB	NS	R	R	I+	I+	R	R	R	NR	R	S	R	NT	XDR
AB217-VUB	R	R	R	I+	I	R	I	S	R	R	S	R	R	PDR
AB219-VUB	R	R	R	I+	I+	R	R	R	NR	R	S	R	NT	XDR
AB220-VUB	R	R	R	I+	I	R	R	NS	NR	R	S	R	NT	XDR
AB222-VUB	R	R	R	I+	I	R	R	I	S	R	S	R	NT	XDR
AB224-VUB	R	R	R	I+	I+	R	R	R	R	R	S	S	NT	XDR
AB226-VUB	R	R	R	I+	I+	R	R	R	I	R	S	S	NT	XDR
AB227-VUB	R	R	R	NR	S	R	S	R	NQ	R	S	S	NT	MDR
AB229-VUB	R	R	R	NR	NR	R	S	R	NQ	R	S	S	NT	MDR
AB231-VUB	NS	R	R	I+	S	R	R	R	R	R	S	R	R	PDR
AB232-VUB	R	R	R	S	S	R	S	R	NR	R	S	S	NT	MDR
AB233-VUB	R	R	R	I+	I	R	NS	NQ	NR	R	S	R	NT	XDR

a AMS, ampicillin/sulbactam; PIP, piperacillin; PIT, piperacillin/tazobactam; CAZ, ceftazidime; ATM, aztreonam; MEM, meropenem; GEN, gentamicin; AMK, amikacin; COL, colistin; CIP, ciprofloxacin; TGC, tigocycline; SXT, trimethoprim-sulfamethoxazole; TET, tetracycline; R, resistant; S, susceptible; I, intermediate; I+, intermediate or resistant (the MICs reached maximum of the kit yet according to CLSI classified as intermediate); NS, nonsusceptible (the triplicates varied in the resistant or intermediate category, yet none was susceptible); NR, nonresistant (the triplicates varied in the intermediate or susceptible category, yet none was resistant); NQ, not qualifiable (the resistant and susceptible phenotypes were detected for the strain within the triplicate); NT, not tested; MDR, multidrug resistant; XDR, extensively drug resistant; PDR, pandrug resistant.

b No breakpoints for these antibiotics are provided by CLSI.

**FIG 4:**
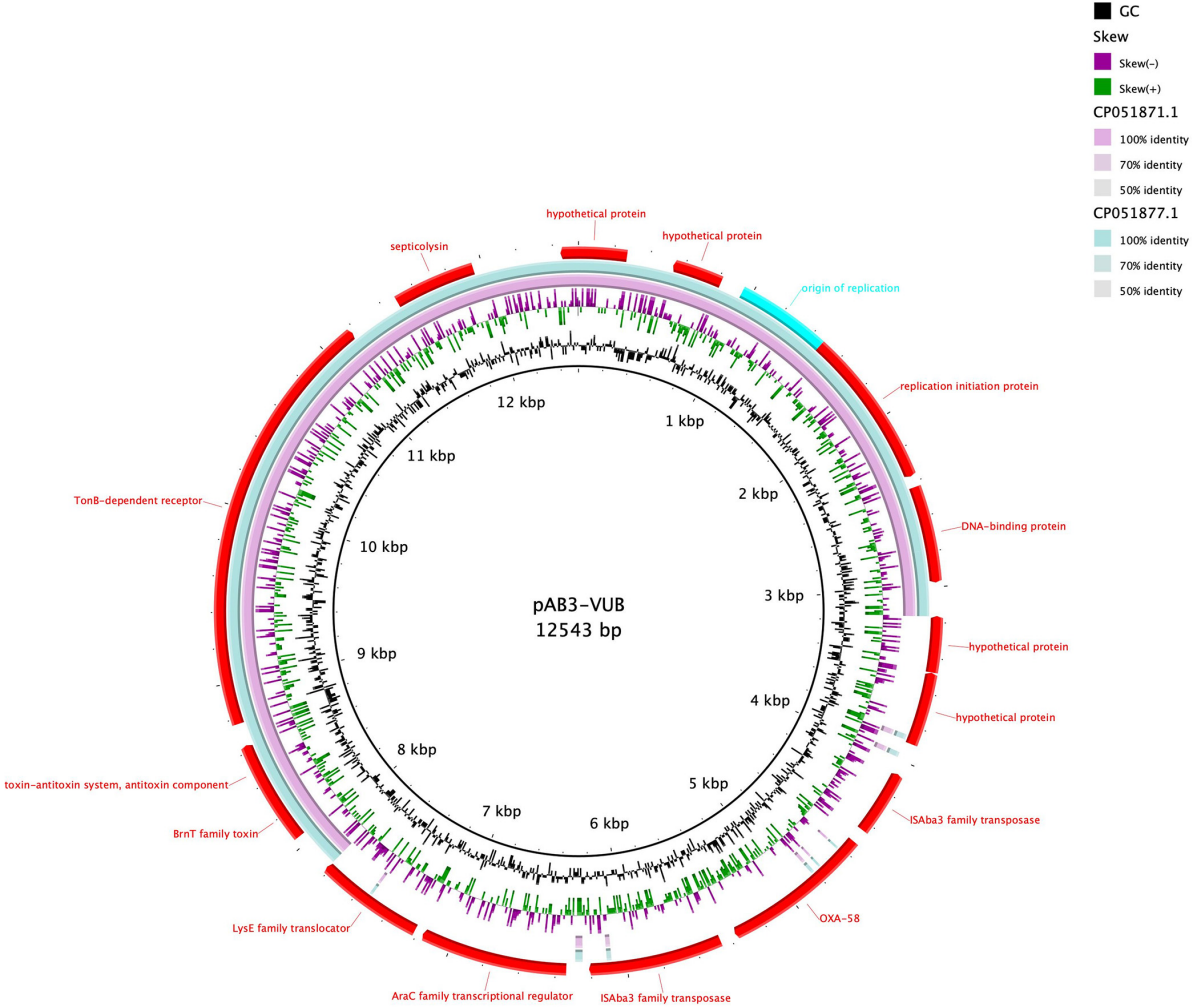
BRIG comparison of pAB3-VUB (reference) originating from Belgian clinical isolates with plasmids pAb-D10a-a_2 (CP051871.1) and pAb-B004d-c_2 (CP051877.1) of Ghanaian origin, showing high nucleotide similarity of the plasmid backbone.

**FIG 5:**
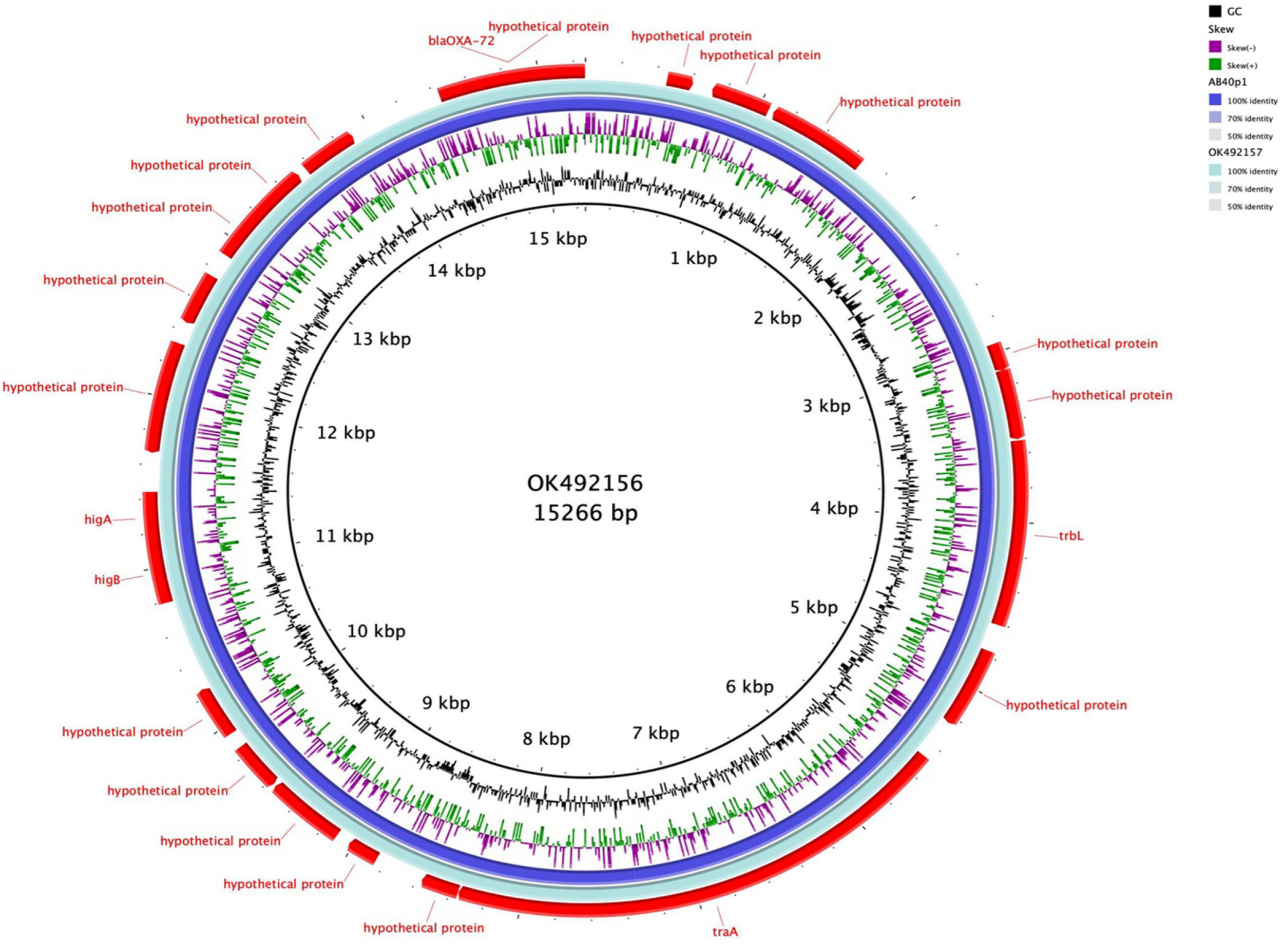
BRIG comparison of p1AB40-VUB with pABCTX2 and pAbCTX11 (OK492156 and OK492157) as a reference, showing a high nucleotide identity of plasmids originating from Belgian isolates to plasmids of French clinical origin.

**FIG 6:**
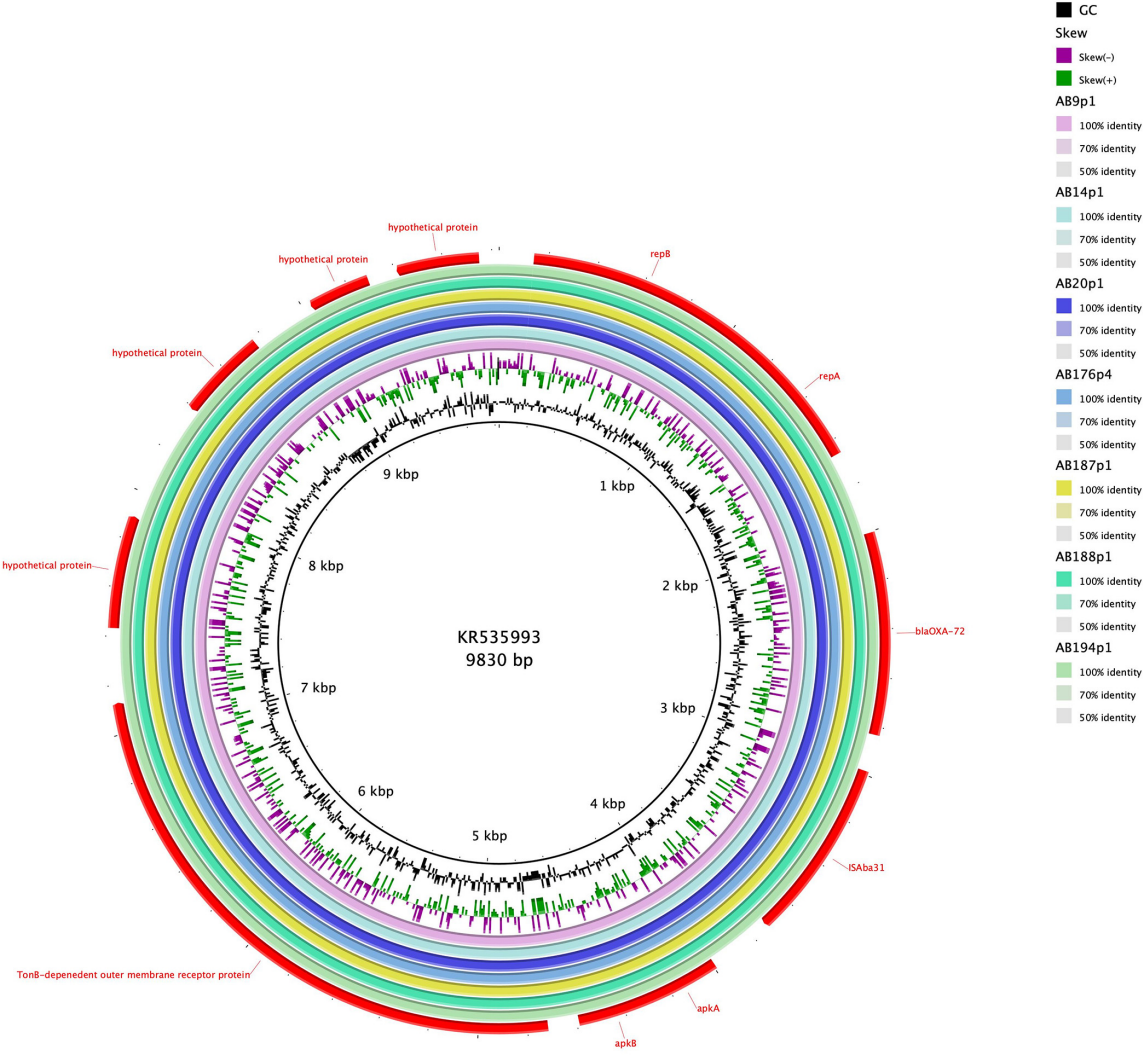
BRIG comparison of p1AB9-VUB, p1AB14-VUB, p1AB20-VUB, p4AB176-VUB, p1AB187-VUB, p2AB188-VUB, and p1AB194-VUB with pA105-2 (KR535993) as a reference, showing a 100% nucleotide identity of plasmids originating from Belgian isolates to pA105-2, which is of Swedish origin.

Global distribution of Tn*2006* and Tn*2008* has been observed before, and Tn*2008* is identified on conjugative plasmids (11). Isolate AB212-VUB carries chromosomally encoded *bla*_OXA-58_ within a composite transposon of IS*18* (IS*30* family). The composite transposon of AB212-VUB is preceded by IS*Aba125*. However, isolate AB3-VUB carries *bla*_OXA-58_ on a plasmid of 12,543 bp resembling the backbone of pAb-D10a-a_2 and pAb-B004d-c_2 (Fig. 4) from Ghana (GenBank accession no. CP051871.1 and CP051877.1, respectively), except that these two plasmids do not carry any antibiotic resistance genes. Seven out of eight *bla*_OXA-72_ genes are associated with a single copy of IS*Aba31*, while *bla*_OXA-72_ of AB40-VUB is not associated with any insertion sequence. The *bla*_OXA-72_ genes for all eight isolates are plasmid encoded, with the plasmid in strain AB40-VUB showing a high sequence similarity to plasmids pABCTX2 and pAbCTX11 (OK492156 and OK492157), from French clinical isolates obtained in 2015 and 2017, respectively (Fig. 5), while AB40-VUB was obtained in 2014. The plasmids in the other seven isolates carrying *bla*_OXA-72_ genes were identical to plasmid pA105-2 (KR535993), which was isolated in Sweden in 2013 (12) and which is 99% similar to pMAL-1 (13) (Fig. 6), suggesting epidemic potential for this plasmid. The gene *bla*_NDM-1_ carried by isolate AB177-VUB is chromosomally encoded within an isoform of the Tn*125* transposon consisting of IS*Aba125* truncated by IS*Aba14*, similar to the case of NDM-1 producing A. baumannii of ST85 from Tunisia (14). Three isolates (AB21-VUB, AB169-VUB, and AB179-VUB) do not carry any carbapenemase-encoding genes, confirmed by their susceptible phenotype (Fig. 1, Table 2, and Table S1). A complete overview of the localization of the carbapenemase-encoding genes and associated mobile elements can be found in Table 1. We also detected high prevalence of genes encoding resistance to aminoglycosides [*aac*(*3*)*-Ia* (*n* = 14), *aph*(*3″*)*-Ib* (*n* = 26), *aph*(*6*)*-Id* (*n* = 26), and *aph*(*3′*)*-Ia* (*n* = 26)], sulfonamides (*sul1* [*n* = 24] and *sul2* [*n* = 14]), and tetracycline [*tet*(*B*) (*n* = 24)] but also to different types of antimicrobials (Fig. 1).

We identified 20 different and 2 novel sequence types (STs) using the Oxford scheme (Fig. 1) and 9 different STs using the Pasteur scheme, for which ST2 is predominant (*n* = 25), followed by ST636 (*n* = 6), ST1 (*n* = 3), ST85 (*n* = 2), ST78 (*n* = 2), and then ST604, ST215, ST158, and ST10 (*n* = 1 isolate each) (Fig. 1). ST2 and ST1, previously described as clinically relevant groups, were among the most widely disseminated STs in the complete and draft genomes currently available in the databases. The two newly assigned STs (Oxford) ST2454 for AB21-VUB (*gltA*-2, *gyrB*-21, *gdhB*-12, *recA*-32, *cpn60*-26, *gpi*-142, *rpoD*-5) and ST2455 for AB179-VUB (*gltA*-1, *gyrB*-1, *gdhB*-13, *recA*-12, *cpn60*-94, *gpi*-16, *rpoD*-2) are now deposited in the PubMLST database. A. baumannii ST2 and ST1 account for 71% of all genomes sequenced from publicly available databases (15). Isolates of the predominant ST2 are widely distributed in Belgium, carrying a broad variety of acquired antimicrobial resistance (AMR) genes, including mainly *aph*(*3″*)*-Ib* (*n* = 23), *aph*(*6*)*-Id* (*n* = 23), *bla*_OXA-23_ (*n* = 22), and *tet*(B) (*n* = 22). A similar AMR profile and phylogenetic relatedness to the ST2 group were detected for AB226-VUB (Fig. 1), which belongs to the rare ST604, which was first identified in Egypt (16). Isolates representing other less frequently detected STs (one isolate per ST) are of ST215 (AB231-VUB) and ST158 (AB32-VUB). Two isolates of ST85 (AB177-VUB and AB186-VUB) and two isolates of ST78 (AB21-VUB and AB40-VUB) did not cluster with any of the major branches ST2, ST1, and ST636 in the phylogenetic tree (Fig. 1), showing their distinct genetic backgrounds.

Concerning the geographical repartition of the different ST identified in our collection, we have detected six isolates of ST636 which have been described to cause outbreaks within hospital settings in Serbia and Colombia (17, 18). A. baumannii ST215 has been common in Thailand since 2010 (19), while GES-producing A. baumannii ST158 caused an outbreak in a Tunisian neonatal unit and was linked to a GES-producing clone from the Middle East; it has also been identified in Denmark (20). ST78 (AB21-VUB and AB40-VUB) was recently detected in Russia as an uncommon clone known as “Italian clone.” Indeed, it was reported from several Italian hospitals in 2010, and since then, it has been detected from other Mediterranean countries, the United States, Germany, Kuwait, and French Guiana, pointing toward successful global dissemination (21). ST85 is represented by two isolates (AB177-VUB and AB186-VUB), yet only AB177-VUB possesses both *bla*_NDM-1_ and *bla*_OXA-94_. A. baumannii ST85 possessing the *bla*_NDM-1_ and *bla*_OXA-94_ genes was previously detected in France, Algeria, Turkey, Syria, Tunisia, and, recently, also Spain (22). AB186-VUB possesses *bla*_OXA-94_ but not *bla*_NDM-1_, pointing toward geographical unrelatedness of AB177-VUB and AB186-VUB.

**FIG 7:**
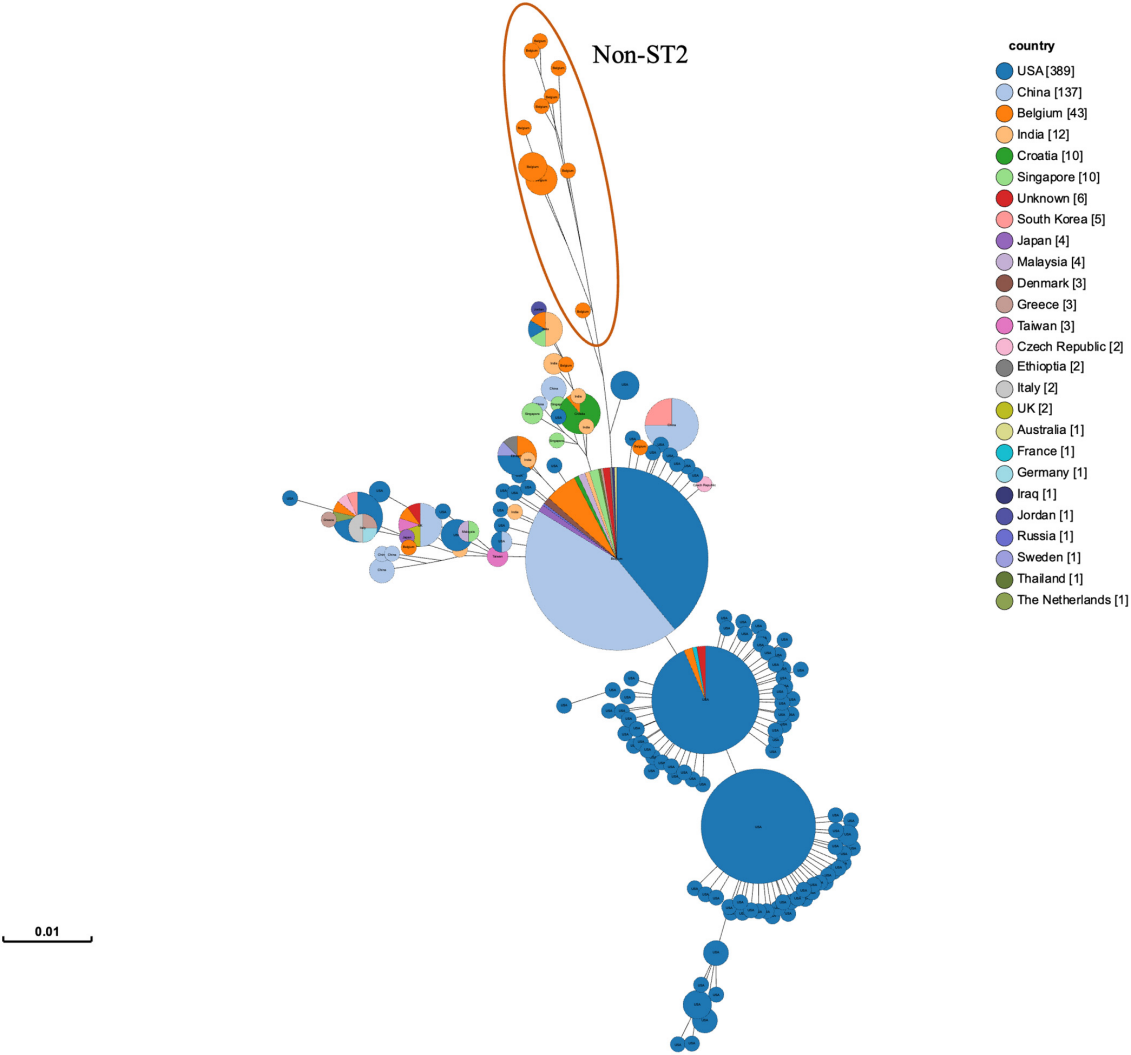
MST tree of 43 clinical isolates of A. baumannii from Belgium compared to 603 whole-genome sequences of A. baumannii ST2 (Pasteur) from BIGSdb, colored according to country. Branches under 0.00006 have been collapsed.

The comparison of our 43 A. baumannii isolates from this study with 603 whole-genome sequences of A. baumannii ST2 obtained from BIGSdb showed great variety and a distribution of A. baumannii ST2 across the world (Fig. 7 and Fig. S1 and S2). The relatedness of the isolates was assessed based on single-nucleotide polymorphisms (SNPs) in coding regions, with threshold for a clonal isolate set for ≤10 as described before (23, 24). The complete overview of SNP distances can be found in the SNP matrix in Table S2. Only two isolates (AB189-VUB and AB222-VUB) met this criterion of the relatedness. Isolate AB189-VUB can be clonally linked to 31 genomes of A. baumannii from the United States (*n* = 28) and France (*n* = 1) and of unknown origin (*n* = 2) (Table S2). On the other hand, strain AB222-VUB is clonally related to 36 genomes of A. baumannii ST2, from the United States (*n* = 33) and France (*n* = 1) and of unknown origin (*n* = 2) (Table S2). While the majority of clonally related strains were the same for both AB189-VUB and AB222-VUB, two isolates from the United States were specific for AB189-VUB and nine isolates from the United States were specific for AB222-VUB. The complete overview on the origin of publicly available A. baumannii ST2 (Pasteur) from BIGSdb can be found in Table S3.

The fact that these isolates were detected in Belgium points toward their persistence and successful global dissemination, especially in the case of the clones of AB189-VUB and AB222-VUB, which were detected in the genomes of isolates from the United States and France. However, specific routes of transmission cannot be established in this study, and certain bias of the sequencing capacity of each country is also present.

Since none of the isolates harbored the *mcr* gene, we have explored the genetic background of the isolates for mutations in the two-component lipid A-encoding system *pmrAB*. Only isolate AB173-VUB harbored a substitution in *pmrB*^T235I^ while *pmrB*^T235N^ was described to provide resistance to colistin (25), possibly providing the same colistin-resistant phenotype. We have also examined interruption of the Lpx pathway as a possible cause of colistin resistance; however, the genes *lpxACD* were intact, suggesting that this mechanism was not present in the studied set of isolates. However, other factors such as outer membrane asymmetry or efflux pumps might be involved (26). Recent data from 30 European countries showed that 4% of the tested CRAb isolates were resistant to colistin, with the majority originating in southern Europe (Greece and Italy) (4).

Despite a limited number of isolates in this study, our findings provide important epidemiological data for Belgium, since most of the data related to MDR A. baumannii and CRAb in Belgium were published before 2010 (27–29).

The data described here provide an insight in the genotype and phenotype of MDR, XDR, and PDR A. baumannii from Belgian hospitals. Carriage of determinants of resistance to carbapenems on mobile genetic elements such as plasmids enables horizontal gene transfer, for which several A. baumannii isolates are naturally competent, and further spread of carbapenem resistance. Our study demonstrates the wide distribution of internationally disseminated MDR, XDR, and PDR clones of A. baumannii in Belgian health care facilities and also shows their detection throughout several years in America, especially in the United States. These strains pose a serious health issue to patients, especially those admitted to high-risk wards such as the intensive care units, and have the potential to cause nosocomial infections and difficult-to-control outbreaks.

## MATERIALS AND METHODS

### Bacterial isolates.

A collection of 43 nonredundant clinical A. baumannii isolates (Fig. 1) collected across Belgium was provided by the National Reference Center (NRC) Laboratory for Antibiotic-Resistant Gram-Negative Bacilli (CHU UCL Namur, Yvoir, Belgium), which acquired these isolates to confirm and characterize carbapenem resistance mechanisms. All isolates were confirmed as A. baumannii by matrix-assisted laser desorption ionization–time of flight (MALDI-TOF) mass spectrometry (MALDI Biotyper; Bruker Daltonics).

### Antimicrobial susceptibility testing.

The antimicrobial susceptibility and MICs were determined by broth microdilution method using the MIKRO-LA-TEST MIC NEFERM kit according to the manufacturer’s instructions (Erba Lachema, Brno, Czech Republic) in triplicates. The results were evaluated according to the CLSI (30, 31). In order to evaluate multidrug-resistant (MDR), extensively drug-resistant (XDR), and pandrug-resistant (PDR) phenotypes (32), susceptibility to tetracycline was tested too, using Etest (bioMérieux). The susceptibility to tetracycline was tested only in isolates carrying the *tet*(*B*) gene, for which resistance to tetracycline would alter the phenotype from XDR to PDR. Two isolates (AB14-VUB and AB189-VUB) not carrying genes conferring resistance to tetracycline were included as a negative controls.

### Whole-genome sequencing.

A total of 43 clinical isolates were subjected to whole-genome sequencing (WGS) using short-read (Illumina) and long-read sequencing (Nanopore) and *de novo* assembly of the draft genomes. The subcultured isolates were used for DNA extraction and following independent sequencing and bioinformatical analyses. Seeing the high genomic dynamics of A. baumannii bacteria, we followed the nomenclature in the field (33) by renaming the subcultured strains by adding “-VUB,” although these strains are *a priori* identical or very similar.

For the short-read sequencing, the genomic DNA was extracted using the phenol-chloroform method. Stationary-phase bacteria (2 mL) at an optical density at 600 nm (OD_600_) of 4 were centrifuged for 1 min at 12,000 × *g* and resuspended in 200 μL of breaking buffer (2% Triton X-100, 1% SDS, 100 mM NaCl, 10 mM Tris [pH 8.0], 1 mM EDTA [pH 8.0]). Then an ~200-μL volume of glass beads and 200 μL of phenol-chloroform were added and vortexed at low speed for 30 s. A 200-μL volume of TE buffer (10 mM Tris and 1 mM EDTA [pH 8.0]) was added, mixed, and centrifuged for 5 min at 7,000 × *g*. The supernatant was transferred into a new Eppendorf tube and 400 μL of phenol-chloroform was added. After centrifugation, the aqueous layer was transferred to a new recipient tube and 1 mL of 100% ethanol was added, mixed, and centrifuged for 3 min at 12,000 × *g*. The supernatant was then removed, the pellet was resuspended with 400 μL of TE buffer, and 30 μL of 1-mg/mL RNase was added. After incubation for 15 min at 37°C, 10 μL of 4 M ammonium acetate was mixed, then 1 mL of ethanol 100% was added. After centrifugation (5 min at 12,000 × *g*), the pellet was resuspended in 100 μL of TE buffer and the final DNA concentration was determined by spectrophotometry.

The sequencing libraries were prepared using Nextera XT and subjected to 2 × 250-bp paired-end sequencing on MiSeq (Illumina) using V3_600 kit. The fastq files were generated and demultiplexed using bcl2fastq (Illumina).

The DNA for long-read MinION (Oxford Nanopore Technologies [ONT]) sequencing was extracted using Genomic-tip 100/G (Qiagen, Hilden, Germany). The long-read sequencing libraries were prepared using a 1D ligation barcoding kit (SQK-LSK109 and EXP-NBD104; ONT, Oxford, UK). Samples were quality controlled using Qubit (double-stranded DNA [dsDNA] broad range (BR) chemistry; Thermo Fisher Scientific) and Fragment Analyzer (Agilent Technologies; using a DNF-464 kit). The average size of the fragments was 45 to 70 kb. Samples were equimolarly pooled and 12 samples were run per sequencing run which was always 2× reloaded. MinION flow cells had a minimum of 1,200 sequenceable pores at the start, and initial loading was approximately 35 fmol followed by 2 reloads each after 24 h of sequencing. The sequencing was performed on a MinION Mk1b instrument (ONT) using R9.4.1 (FLO-MIN106) flow cells.

### Sequence data analysis.

The long-read sequences were demultiplexed and base called using Guppy v3.2.2 and subsequently were adaptor, quality (Q ≤ 13), and length (5,000 bp) trimmed using Porechop v0.2.2 (https://github.com/rrwick/Porechop) and NanoFilt v2.8.0 (34), respectively. The short reads (BioProject PRJNA734485) were used to polish the long reads employing Ratatosk v0.7.0 (35). The corrected reads were then assembled using Unicycler v0.4.8 (36).

### Genotypic characterization.

The assembled draft genomes were subjected to multilocus sequence typing (MLST) using mlst (https://github.com/tseemann/mlst) employing the PubMLST database (37) based on the Pasteur and Oxford schemes. Two isolates (AB21-VUB and AB179-VUB) were of a novel ST^Oxford^ and were deposited to PubMLST database and assigned a new ST. The resistance genes were detected using ABRicate (https://github.com/tseemann/abricate) employing ResFinder (38) with a 95% threshold for both identity and query coverage. The point mutations were characterized using the BLAST algorithm and Geneious R9 (Biomatters, New Zealand). The genetic environment was assessed using Mobile Element Finder by the Center for Genomic Epidemiology (39).

### Phylogenetic analysis.

The maximum likelihood tree depicting the relatedness of the isolates was constructed from assembled draft genomes using precited open reading frames obtained by Prokka (40) as an input for the core genome alignment created using Roary (41). RAxML (42) was used for calculation of the phylogenetic tree using general time reversible with optimization of substitution rates under the GAMMA model of rate heterogeneity method supported by 500 bootstraps. The phylogenetic tree was visualized and completed with metadata in iTOL (43).

### Comparison with publicly available genomes.

The most clinically relevant isolates belonging to worldwide-spread ST2 (Pasteur scheme) were compared to genomes of A. baumannii ST2 available in BIGSdb (44). The search was performed on 14 June 2022 and resulted in 607 hits for A. baumannii ST2 containing sequencing data. Out of 607 entries, 4 were excluded after not passing the ST verification using *in silico* MLST by mlst (https://github.com/tseemann/mlst). All 43 isolates from our study were involved. The annotation and core genome alignment were performed using Prokka and Roary as described above. SNPs were extracted from the core genome alignment using snp-sites (https://github.com/sanger-pathogens/snp-sites), and the phylogenetic tree was constructed using RAxML under the GTRGAMMA model supported by 100 bootstraps. The minimum spanning tree (MST) was visualized using GrapeTree (45). The relatedness of the isolates was assessed based on core genome SNP count obtained using snp-dists (https://github.com/tseemann/snp-dists) with a cutoff value of ≤10 for clonal relationship as described before (23, 24).

### Data availability.

The draft and complete assemblies with the short- and long-read sequencing reads were deposited in GenBank under BioProject PRJNA734485, PRJNA701627, and PRJNA798866.
