# Supplementary material for: Genomic Analysis of a Strain Collection Containing Multidrug-, Extensively Drug-, Pandrug-, and Carbapenem-Resistant Modern Clinical Isolates of Acinetobacter baumannii
Source: Antimicrob Agents Chemother. 2022 Aug 15;66(9):e00892-22. doi: 10.1128/aac.00892-22 (PMC9487538; doi:10.1128/aac.00892-22)
Supplement: Supplemental file 1 — Fig. S1 and S2. Download aac.00892-22-s0001.pdf, PDF file, 0.8 MB [file aac.00892-22-s0001.pdf]

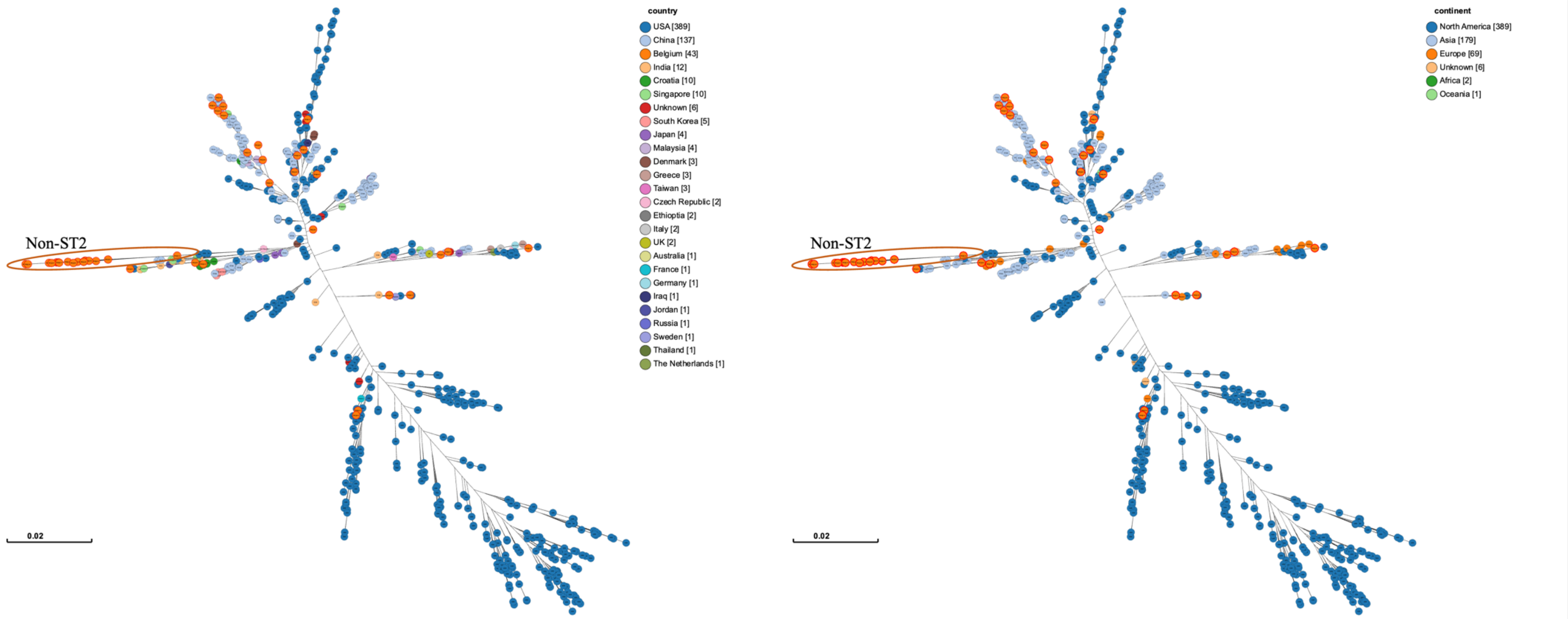

Supplementary Figure 1: A MST trees of 43 clinical siolates of *A. baumannii* from belgium compared to 603 WGS of *A. baumannii* ST2 (Pasteur) from BIGSdb, coloured according do country (left) and continent (right) of origin. Dynamic Visualization in GrapeTree, logarithmic scale.

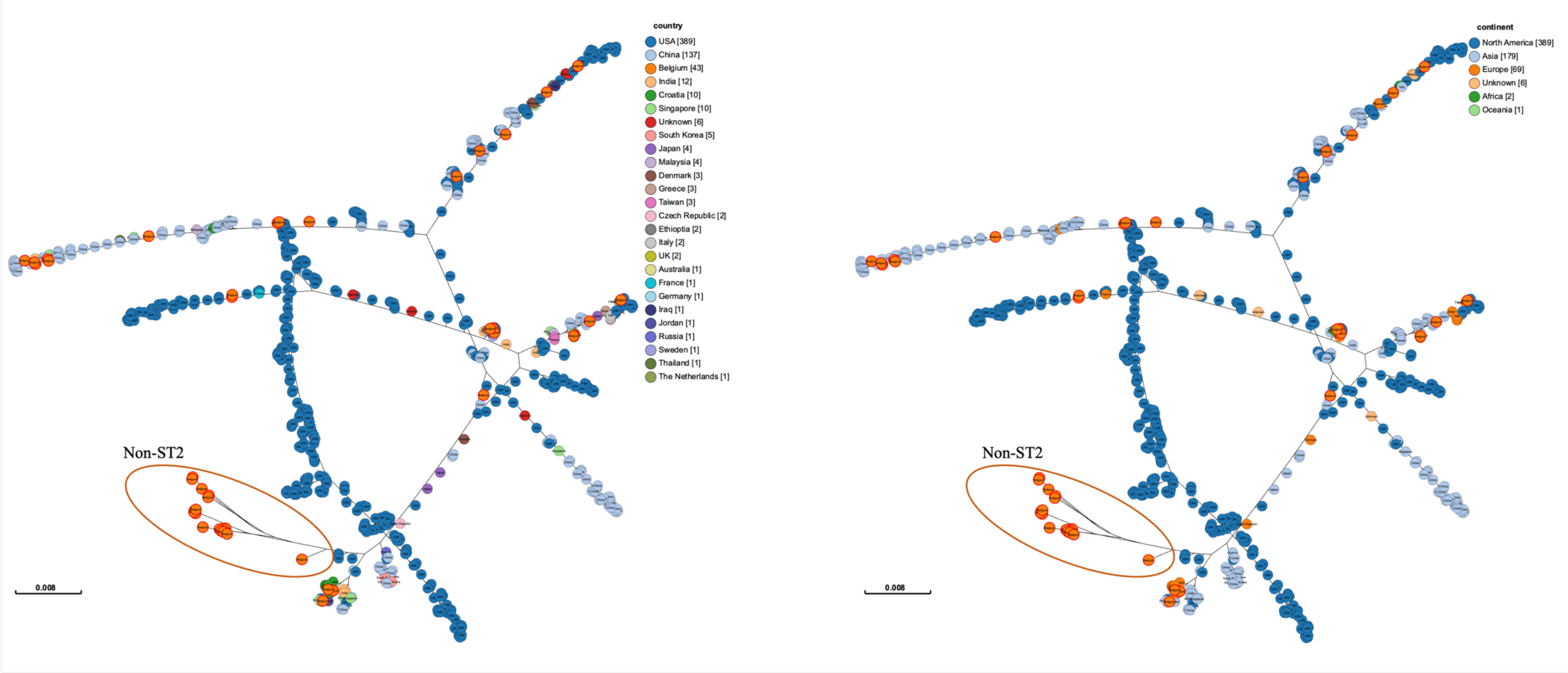

Supplementary Figure 2: A MST trees of 43 clinical siolates of *A. baumannii* from belgium compared to 603 WGS of *A. baumannii* ST2 (Pasteur) from BIGSdb, coloured according do country (left) and continent (right) of origin. Dynamic visualization in GrapeTree.
